# Pros and Cons of Audience Response Systems in the Education of Health Professionals

**DOI:** 10.15694/mep.2019.000182.1

**Published:** 2019-09-24

**Authors:** Ryan Denkewicz

**Affiliations:** 1 Frank Netter School of Medicine at Quinnipiac University

**Keywords:** Audience response systems, ARS, clickers, learning outcomes, interactive learning, active learning

## Abstract

This article was migrated. The article was marked as recommended.

Well over 1000 articles have been written on the subject of Audience Response Systems in the education of health professionals. These studies have involved the students at the undergraduate, graduate and continuing professional education levels. From the articles found, a subset of over 120 articles of ARS use in health professional education was reviewed. A holistic view of the results in these studies showed that there are clear benefits and drawbacks to ARS use from the perspective of the students and teachers. Benefits were found to fit ten (10) distinct categories while drawbacks fit into six (6) discrete categories. This paper summarizes and discusses the benefits (i.e. pros) and drawbacks (i.e. cons) of the use of ARS in the educational process of health professionals.

## Introduction

Audience Response System (ARS) technologyhas been used over the last several decades as another modality of teaching to enhance learning outcomes ( Boyle, 2006; Keengwe, 2007). The technology was first developed by IBM in the 1960s.One of its first uses was in the area of market research, most notably to collect anonymous survey data from consumers previewing unreleased Hollywood films. Academically, ARS was first seen in the 1960s at Cornell and Stanford universities ( Kay and LeSage, 2009; Nelson *et al.,* 2012). More recently, ARS technology has been gaining popularity in the classroom ( Han, 2014) including its more frequent use by medical and dental schools, medical residency programs and studies in the biological sciences and health professions. The collective body of research on ARS has highlighted both the attendant benefits and drawbacks associated with this new pedagogical tool. While some studies have demonstrated a positive statistical correlation between ARS use and knowledge retention and exams scores ( Crouch and Mazur, 2001; Bunce, VandenPlas and Havanki, 2006; Mains *et al.,* 2015; Subramanian *et al.,* 2011; Benson *et al.,* 2017; Stoddard and Piquette, 2010; Nelson *et al.,* 2012; Berry, 2009; Alexander *et al.,* 2009; Hettinger *et al.,* 2014; Nosek *et al.,* 2006; Slain *et al.,* 2004), others have found the use of ARS to be disruptive to the classical didactic teaching approach ( Clausen, Alkhateeb and Singh-Franco, 2012; Connor, 2009) and too cumbersome on the instructor ( Abate, Gomes and Linton, 2011; Arneja *et al.,* 2009; Cain and Robinson, 2008; De Gagne, 2011; DeBourgh, 2008; Zurmehly and Leadingham, 2008).

This paper summarizes the pros and cons of ARS use as cited by various studies, particularly those performed in the area of health professional education, including continuing education and conferences for physicians along with some undergraduate studies in biological and health sciences (see Table 1). It is hoped that the review and analysis performed here accomplishes at least two objectives: 1) provides a more complete picture of the potential for ARS technology, and, 2) provides insights to teaching professionals deciding if ARS technology should be incorporated into their specific teaching regimen.

**Table 1.** Articles in the Health Profession on Audience Response Systems

Student Classification	# of Papers Reviewed
Medical Students	48
Medical Residents	10
Physicians	13
Nursing & Pharmacy	27
Veterinary & Dental Medicine	15
Biological & Health Sciences	15

### What is ARS Technology?

ARS technology, in its most simplistic form, consists of handheld devices (called clickers) that are remotely connected to a hardware device that contains software which enables the collection and display of student responses in the classroom via the instructor’s computer ( Collins, 2008; Thampy and Ahmad, 2014; Vana *et al.,* 2011). ARS goes by other names including personal response system, interactive voting system, electronic voting system, student response system, interactive student response systems, group response system, classroom response system, immediate response system, classroom performance system and clickers ( Hunsu, Adesope and Bayly, 2016). A very common implementation of the technology is for the teacher to display a multiple choice question in the classroom (e.g. using a projector and a program such as Microsoft Powerpoint), with the students using their clicker devices to select what they believe is the correct answer. The ARS system collects the responses and displays them in a tabular or graphic format. The handheld clickers can be registered online such that each device is tied to a specific student. This is desirable if the ARS system is being used to confirm class attendance and/or record student performance. Alternatively, systems can be configured whereby the student submits responses using any wireless network-enabled device (e.g. laptop, cellphone). The use of ARS can replace the more conventional teacher-student interaction whereby the teacher poses a question and then calls upon a single student for an answer. Here, the entire student body is involved with the preservation of anonymity.

ARS can be used to achieve a number of educational objectives. These include: ( Dufresne *et al.,* 1996):

• Aiding the instructor in identifying the students’ understanding
• Aiding the instructor in evaluating his/her own teaching performance
• Increasing the students’ knowledge of difficult concepts
• Increasing the students’ knowledge of the instructors expectations
• Enhancing the students’ awareness of their own understanding
• Providing a ‘springboard’ for class debate and discussion
Each of these objectives is valuable in its own right, but when taken together, they potentially represent a significant new tool in education. For this reason, ARS is worthy of critical review and analysis.

## Methodology

Hundreds of research papers were located on PubMed, EMBASE, SCOPUS, Cochrane Library, CINAHL, ERIC using the keywords, audience response systems, personal response stations, interactive voting systems, electronic voting systems, student response systems, interactive student response systems, group response systems, and clickers. The research studies located were then narrowed to the field of health professional education. This included studies performed in undergraduate college level biology and anatomy classes, nursing, dental, veterinary and medical school curricula, as well as residency programs and continuing educational classes for physicians and other health professionals. Inclusion of research articles found beyond the scope of the health profession, would have led to studies numbering well over 1000.

The pros and cons of the use of ARS technology, from both the perspective of the students and teachers, were captured and then compiled into distinct categories.

## Discussion

A review of over 120 research articles on the use of ARS technology in the education of health professionals led to a myriad of opinions, attitudes and facts regarding the benefits and drawbacks to ARS in the educational process. When viewed holistically, the perceived and real benefits (i.e. pros) and drawbacks (i.e. cons) cited in the research were found to fit a distinct set of categories as shown in Table 2.

### The Pros

Ten distinct categories of benefits were noted in the compilation of the research studies (see Table 2). These are discussed, in turn, below.

**Table 2.** Pros and Cons of Audience Response Systems

Pros	References
**Increases student Engagement/Attention**	Abate *et al.,* 2011; Addison *et al.,* 2009; Arneja *et al.,* 2009; Baker *et al.,* 2017; Barbour 2008; Beaumont *et al.,* 2017; Benson *et al.,* 2016; Berry 2009; Caldwell 2007; Chaudhry 2011; Clauson *et al.,* 2012; Connor 2009; Copeland *et al.,* 2000; De Gagne 2011; Dhaliwal *et al.,* 2015; Doucet *et al.,* 2009; Fies 2005; Fitzpatrick *et al.,* 2011; Freeman and Dobbie 2008; Gauci *et al.,* 2009; Graeff *et al.,* 2011; Hatch *et al.,* 2005; Ioannou and Artino 2007; Johnson 2005; Kay and Lesage 2009; Kung *et al.,* 2012; Latessa and Mouw 2005; Llena *et al.,* 2015; Mains *et al.,* 2015; Mastoridis and Kladidis 2010; Meedzan and Fisher 2009; Miller *et al.,* 2003; Mostyn *et al.,* 2012; Petit *et al.,* 2014; Pradhan *et al.,* 2005; Premkumar and Coupal 2008; Preszler *et al.,* 2007; Uhari *et al.,* 2003
**Improves Learning outcomes**	Abate *et al.,* 2011; Alexander *et al.,* 2009; Arneja *et al.,* 2009; Baker *et al.,* 2017; Brady and Forest 2018; Brady *et al.,* 2013; Cain and Robinson 2008; Caldwell 2007; Chaudhry 2011; Devitt 2012; Dhaliwal *et al.,* 2015; Doucet *et al.,* 2009; Duggan *et al.,* 2007; Elashvili *et al.,* 2008; Freeman and Dobbie 2008; Goldberg *et al.,* 2006; Grimes *et al.,* 2010; Guse and Zobitz 2010; Hettinger *et al.,* 2014; Hoyt *et al.,* 2010; Hunsu *et al.,* 2015; Jelsing *et al.,* 2007; Johnson 2005; Kay and Lesage 2009; Latessa and Mouw 2005; Leidner and Jarvenpaa 1995; Liu *et al.,* 2010; Lymn and Mostyn 2010; Mahon *et al.,* 2018; Mains *et al.,* 2015; Mastoridis and Kladidis 2010; Meedzan and Fisher 2009; Mostyn *et al.,* 2012; Palmer *et al.,* 2005; Pradhan *et al.,* 2005; Premkumar and Coupal 2008; Preszler *et al.,* 2007; Rubio *et al.,* 2008; Schackow *et al.,* 2004; Schlegel and Selfridge 2014; Uhari *et al.,* 2003
**Provides focus for Study**	Abate *et al.,* 2011; Alexander *et al.,* 2009; Arneja *et al.,* 2009; Benson *et al.,* 2016; Cain and Robinson 2008; Caldwell 2007; Copeland *et al.,* 2000; Crouch and Mazur 2001; De Gagne 2011; Draper *et al.,* 2002; Egelandsdal and Krumsvik 2015; Fitch 2004; Gauci *et al.,* 2009; Halloran 1995; Hatch *et al.,* 2005; Hudson and Bristow 2006; Ioannou and Artino 2007; Johnson 2005; Leung *et al.,* 2013; Mostyn *et al.,* 2012; Pradhan *et al.,* 2005; Premkumar and Coupal 2008; Knight and Wood 2005; Lymn and Mostyn 2010; Robertson 2000; Schlegel and Selfridge 2014; Simpson and Oliver 2007; Stuart *et al.,* 2004; Wait *et al.,* 2009
**Realtime Feedback for Teacher**	Abate *et al.,* 2011; Akl 2010; Alexander *et al.,* 2009; Arneja *et al.,* 2009; Beatty *et al.,* 2006; Beaumont *et al.,* 2017; Benson *et al.,* 2016; Brady and Forest 2018; Bruff 2015; Cain and Robinson 2008; Caldwell 2007; Chaudhry 2011; Connor 2009; De Gagne 2011; Draper *et al.,* 2002; Duggan *et al.,* 2007; Efstathiou and Bailey 2012; Egelandsdal and Krumsvik 2015; Fissell 2016; Fitch 2004; Fitzpatrick *et al.,* 2011; Fujikura *et al.,* 2013; Gauci *et al.,* 2009; Gousseau *et al.,* 2016; Guse and Zobitz 2010; Harvey 2015; Hoyt *et al.,* 2010; Hudson and Bristow 2006; Ioannou and Artino 2007; Kay and Lesage 2009; Kennedy and Cutts 2005; Kung *et al.,* 2012; Leeds *et al.,* 2018; Llena *et al.,* 2015; Lymn and Mostyn 2010; Mastoridis and Kladidis 2010; Menon *et al.,* 2004; Mostyn *et al.,* 2012; Nicol and MacFarlane-Dick 2006; Petit *et al.,* 2014; Revell and McCurry 2010; Schlegel and Selfridge 2014; Simpson and Oliver 2007; Stuart *et al.,* 2004
**Increases Peer Interactivity & Class Participation**	Alexander *et al.,* 2009; Arneja *et al.,* 2009; Baker *et al.,* 2017; Barbour 2008; Beatty *et al.,* 2006; Beaumont *et al.,* 2017; Beekes 2006; Brady and Forest 2018; Caldwell 2007; Dhaliwal *et al.,* 2015; Doucet *et al.,* 2009; Draper *et al.,* 2002; Efstathiou and Bailey 2012; Fissell 2016; Fitzpatrick *et al.,* 2011; Gousseau *et al.,* 2016; Horowitz 1988; Hudson and Bristow 2005; Jackson and Trees 2003; Knight and Wood 2005; Mahon *et al.,* 2018; Naismith and Steinert 2001; Streeter and Rybicki 2006; Wood 2004
**Provides Student Anonymity**	Beekes 2006; Cain and Robinson 2008; Chaudhry 2011; Clauson *et al.,* 2012; Connor 2009; De Gagne 2011; Draper and Brown 2004; Fies 2005; Gauci *et al.,* 2009; Guse and Zobitz 2010; Ioannou and Artino 2007; Jones *et al.,* 2001; Kung *et al.,* 2012; Mahon *et al.,* 2018; Mostyn *et al.,* 2012; Stuart *et al.,* 2004
**Easy to use**	Alexander *et al.,* 2009; Beaumont *et al.,* 2017; Benson *et al.,* 2016; Cain and Robinson 2008; Caldwell 2007; Llena *et al.,* 2015; Miller *et al.,* 2003; Parsons 2005; Pradhan *et al.,* 2005
**Enjoyable to use**	Addison *et al.,* 2009; Akl 2010; Arneja *et al.,* 2009; Barbour 2008; Doucet 2009; Cain and Robinson 2008; De Gagne 2011; Devitt 2011; Fitzpatrick *et al.,* 2011; Freeman and Dobbie 2005; Graeff *et al.,* 2011; Hudson and Bristow 2006; Ioannou and Artino 2007; Latessa and Mouw 2005; Llena *et al.,* 2015; Lymn and Mostyn 2010; Mains *et al.,* 2015; Mastoridis and Kladidis 2010; Petit *et al.,* 2014
**Encourages Active Learning**	Addison *et al.,* 2009; Alexander *et al.,* 2009; Arneja *et al.,* 2009; Caldwell 2007; Clauson *et al.,* 2012; De Gagne 2011; Draper *et al.,* 2002; Goldberg *et al.,* 2006; Hoyt *et al.,* 2010; Johnson 2005; Kung *et al.,* 2012; Meedzan and Fisher 2009; Meyers and Jones 1993; Premkumar and Coupal 2008; Revell and McCurry 2010; Roschelle 2003; Rubio *et al.,* 2008; Simpson and Oliver 2007
**Improves Student Attendance**	Connor 2009; Gauci *et al.,* 2009; Kay and Lesage 2009; Kung *et al.,* 2012
**Cons**	**References**
**Teacher Prep Time**	Abate *et al.,* 2011; Arneja *et al.,* 2009; Collins 2008; De Gagne 2011; Duggan *et al.,* 2007; Gauci *et al.,* 2009; Kay and Lesage 2009; Stuart *et al.,* 2004
**Cost**	Arneja *et al.,* 2009; Barber and Njus 2007; Cain and Robinson 2008; Collins 2008; De Gagne 2011; Mastoridis and Kladidis 2010; Menon *et al.,* 2004
**Learning Curve for use**	Arneja *et al.,* 2009; Cain and Robinson 2008; Collins 2008; Gauci *et al.,* 2009; Gousseau *et al.,* 2016; Kay and Lesage 2009; Kung *et al.,* 2012
**Technical Malfunctions**	Benson *et al.,* 2016; Cain and Robinson 2008; Collins 2008; De Gagne 2011; Gauci et al 2009
**Reduced Classtime**	Clauson *et al.,* 2012; Collins 2008; Connor 2009; Ioannou and Artino 2007; Kay and Lesage 2009
**Students Object to being Monitored**	Fujikura *et al.,* 2013; Kay and Lesage 2009; Meedzan and Fisher 2009

*Increased Student Engagement & Attention **.*** ARS technology was found in a number of studies to increase student engagement in the classroom ( Abate, Gomes and Linton, 2011; Cain and Robinson, 2008; Hatch, Jenson and Moore, 2005). This result was typically gauged through a post-ARS use survey of students and teachers. Unlike conventional didactic lectures where student involvement may or may not be solicited or required, the use of ARS demands some attention by the student. This is particularly true in those applications of its use when the student is graded based on their participation or when the teacher uses the ARS question format to then stimulate a class discussion. Generally, students willingly engaged with the system, citing the desire to challenge their own knowledge against that of their peers and the enjoyment of the trivia game-like environment ARS provides ( Schlegal and Selfridge, 2014). The anonymity offered by ARS also makes it easier for students to participate without the fear of public speaking or ridicule for not knowing the correct answer ( Cain and Robinson, 2008; Draper and Brown, 2004; Beekes, 2006). Teachers cited a pronounced level of student attention during ARS lectures that was not observed in the absence of ARS ( Arneja *et al.,* 2009; Freeman and Dobbie, 2005; Johnson, 2005; Pradhan, Sparano and Ananth, 2005).

*Improved Learning Outcomes.* Multiple studies (see Table 3) reported a distinct improvement in learning outcomes as measured by test scores, including those of National exams. Subramanian *et al.,* (2011) observed a significant improvement in student learning outcome with ARS compared to the traditional didactic lecture format. Specifically, in a course in Cardiology at Baylor Medical School, the mean score for the control group was 61.7 +/- 2% whereas the mean score for the ARS led group was 86.7+/- 2%. After a 22 day waiting period, the students were reassessed on the same information and the control group scored 55.8% while the ARS led group scored 70.1%. Similarly, Arneja et al (2009), observed statistically higher scores ( *p* = 0.01) with the use ARS in a Plastic Surgery Residence program. However, Arneja *et al.,* (2009) and Tregonning *et al.,* (2012) found the improvement in knowledge retention to be short-lived. Some researchers ( Pradhan, Sparano and Ananth, 2005) have speculated that improvements in learning outcomes are simply a result of the Hawthorne effect. That is, the novelty of the Audience Response System, when used for the first time, creates excitement, increased student attention and enhanced learning outcomes, but such benefits diminish over time.

**Table 3.** Studies Reporting the Improvement in Learning Outcomes

Study	Cited evidence of Improved Learning Outcome
Alexander *et al.,* 2009	Statistically significant ( *p*<0.0001) positive correlation between ARS use and final exam scores over 3 year period with 1st year Medical students in all didactic blocks
Arneja *et al.,* 2009	Cumulative mean test scores were 85% (with ARS) and 75% (without ARS) in Plastic Surgery Resident program
Berry, 2009	Student t-test exam and final exam course grades were significantly higher in a Pediatric nursing course
Cain and Robinson, 2008	Significantly higher test and final exams scores (with ARS) over 2 years in Pharmacy Resident program
Elashvili *et al.,* 2008	Statistically significant ( *p*= 0.002) written test scores on post-lecture quiz for Dental students
Gauci *et al.,* 2007	Significant improvement in all 3 years (p<0.01) in undergraduate Physiology course when ARS was used
Hettinger *et al.,* 2014	In 2 year study, overall performance improved significantly (p= 0.0068) on PRITE testing in Psychiatry Resident program
Hoyt *et al.,* 2010	Students in lower quartile had better exam performance after ARS was implemented in Human Anatomy course over 2 year study period
Hunsu, Adesope and Bayly, 2016	Meta-analysis of 53 articles involving 26,095 students showed ARS use produced small but significant effects on measures of knowledge transfer and final achievement across a myriad of subject matters
Mains *et al.,* 2015	ARS use increased knowledge both immediately after lecture as well as 2 weeks later for 1st year Medical School students *(p*=0.001)
Nosek *et al.,* 2006	Mean test score progressively improved with higher use of ARS in a Hematology/Oncology course for 2nd year Medical School students
Palmer *et al.,* 2005	ARS improved the long term retention of material and provided better understanding of topics discussed for 1st year Medical school students
Pradhan, Sparano and Ananth, 2005	21% improvement in test scores ( *p* = 0.018) for Residency students in OB-GYN
Preszler *et al.,* 2007	The more ARS was utilized the better the students performed across 6 separate Biology classes
Rubio *et al.,* 2008	ARS group had significantly higher learning ( *p*=0.02) and long term retention ( *p*=0.001) scores on post-lecture quizzes and quizzes 3 months later in a Radiology Resident program
Schackow *et al.,* 2004	96% (ARS) versus 61% (non-ARS) post-lecture quiz score for Family Medicine Residents
Schlegal and Selfridge, 2014	Improvement in academic performance was observed in Microbiology, Pharmacology, Pathology and Clinical Medicine
Slain *et al.,* 2004	Students using ARS in Clinical Pharmacokinetics, Medical Literature Evaluation, and Pathophysiology and Therapeutics had better final exam scores ( *p*<0.001, *p*=0.016 and *p*= 0.0002, respectively)
Subramanian *et al.,* 2012	ARS modality demonstrated a significant improvement ( *p* < 0.001) in student learning retention compared to traditional didactic lecture in Cardiology students at Baylor Medical School, both immediately after lecture and after 22 days
Tregonning *et al.,* 2012	Significantly higher immediate post-lecture quiz scores ( *p*<0.001) in an Ob-Gyn course.

A few studies ( Alexander *et al.,* 2009; Schackow *et al.,* 2004; Premkumar and Coupal, 2008; Rubio *et al.,* 2008; Devitt, 2012; Leidner and Jarvenpaa, 1995; Mastoridis and Kladidis, 2010; Meedzan and Kelly, 2009; Palmer *et al.,* 2005) claim that longer term knowledge retention did occur when ARS was employed.

In a dental studies course ( Wenz *et al.,* 2014), it was observed that the greatest performance improvement with ARS benefited the ‘below-average" students.

Other studies showed no measurable improvement in testing scores ( Patterson, Kilpatrick and Woebkenberg, 2010; Stoddard and Piquet, 2010; Addison, 2008; Graeff *et al.,* 2011; Plant, 2007; Welch, 2013). Stoddard and Piquet (2010), for example, observed no measureable difference in exams scores in a medical school Pulmonology class when ARS was used. Similarly, Addison (2008) saw no improvement in class mean composite exam scores in an undergraduate biochemistry course between students taught with clickers than those taught in traditional lectures. Graeff *et al.,* (2011), in a Physician Assistant program, observed no statistical difference in knowledge retention between groups that had traditional lecture formats and those that had used clicker response questions.

*Provides Focus for Study.* Students perceive that the material highlighted by the teacher using ARS, provides a clear indication of the most important areas of study ( Benson *et al.,* 2016; Abate, Gomes and Linton, 2011; Ionone and Aretino, 2007). As such, students did report a belief that ARS helps guide their preparation for class discussion and exams by identifying gaps in their understanding of the presented material (Goose and Obits, 2010; Mostyn, Meade and Lymn, 2012; Schlagle and Selfridge, 2014). In one study at a Urological Conference ( Leung *et al.,* 2013), it was noted that the use of ARS may be able to identify high-yield topics for medical education.

*Real-time Feedback for Teacher.*Audience Response Systems have the unique benefit of providing immediate feedback to the teacher as to the knowledge gaps that exist among students. Well posed questions help illuminate areas of student conceptual misunderstanding ( Duggan, Palmer and Devitt, 2007; Fujikura *et al.,* 2013). ARS immediately provides teachers with the data to know whether or not they are adequately conveying the information. Are more example problems needed? Is a class discussion/debate warranted?Such formative feedback is useful as it affords the teacher the ability to adjust the lecture ‘on the fly’ or reconfigure future lectures, or homework assignments, to address knowledge gaps. This ‘contingent teaching’ or ‘agile teaching’ is responsive to the needs of the students and not driven by a preset download of information determined by the teacher ( Draper & Brown, 2004; Beatty *et al.,* 2006).

Additionally, formative feedback from the ARS approach enables teacher to gauge their own teaching style and performance ( Draper, Cargill and Cutts, 2002). Am I moving too fast through the material? Should I try an alternative means of conveying the concept? Might a specific homework assignment be valuable to solidify the students’ understanding? Through the instructor’s self-reflection catalyzed by the use of ARS, the teaching dynamic changes in that it “moves [the instructor] out from behind the relative safety of the lecture podium to adopt active strategies that shift classroom emphasis away from teachers’ teaching towards students’ participation and learning” ( Allen and Tanner, 2005).

*Increased Peer Interactivity and Class participation.*Inherent in the design of Audience Response Systems is the engagement of all the students in the subject matter being taught through the answering of questions followed by a review of the responses. Some teachers have used the results of the student responses to spark classroom debate over why one answer was better than another ( Alexander *et al.,* 2009; Horowitz, 1988; Cain and Robinson, 2008; Beekes, 2006; Fissell, 2016). Encouraging peer-to-peer interaction is considered an invaluable technique in education ( Mazur, 1997). Harvard professor Eric Mazur first found that “Peer Instruction” is one of the best modes for learning; that is, students were better at clearing up each other’s misconceptions and confusions than the instructor themselves. Wood (2004) demonstrated this peer-to-peer benefit directly in an undergraduate biology class studying molecular genetics. Towards this end, ARS provides a ready platform for classroom discussion and debate over the best answer and alternative viewpoints.

Additionally, Fies (2005) no`tes that the “level of intrinsic motivation [for students] increases with an engaging learning environment in which mastery goals are emphasized and frequent feedback is given.”

*Provides Student Anonymity.* Perhaps one of the greatest barriers to student learning is certain fears that a student may have in a public classroom environment ( Clausen, Alkhateeb and Singh-Franco, 2012; De Gagne, 2011; Fies, 2005; Ioannou and Artino, 2007).Many students readily admit that they choose not to engage in dialogue during class time for fear of:

• Saying something that makes them look ‘stupid’ in front of their peers
• Looking like they are showing off (if they happen to have the correct response)
• Public speaking

These student fears are well documented and are real barriers for many ( Caldwell, 2007; Chaudhry, 2011; Connor, 2009; De Gagne, 2011; Fies, 2005; Gauci *et al.,* 2009; Guse and Zobitz, 2010; Ioannou and Artino, 2007; Kung *et al.,* 2012). However, ARS, by virtue of the anonymity it provides, helps remove these fears. With ARS, students are able to actively participate without the possibility of public embarrassment or scrutiny. This may be particularly helpful to those students who are struggling with the conceptual understanding of the lecture material, as this group might be more inclined not to otherwise participate ( Mostyn, Meade and Lymn, 2012; De Gagne, 2011; Ioannou and Artino, 2007; Connor, 2009).

*Easy to Use.* Clickers have been repeatedly found to be intuitively easy to use ( Beaumont *et al.,* 2017) and no studies were found in which the students found them complicated or confusing.

*Enjoyable to Use.* A nice benefit of ARS is the enhanced student enjoyment ( Halloran, 1995; Mastoridis and Kladidis, 2010; Devitt, 2012; Cain and Robinson, 2008; Arneja *et al.,* 2009; Freeman and Dobbie, 2005; Baker, Francis and Cathcart, 2017; Torbeck, 2007). In fact, some studies reported that student’s class attendance improved with the use of ARS ( Gauci *et al.,* 2009; Kay and Lesage, 2009; Kung *et al.,* 2012). Those students cited the enjoyment of using ARS as the primary reason for increased class attendance. The trivia game-like atmosphere that is created by ARS, makes the classroom dynamic more exciting for many students ( Schlegal and Selfridge, 2014).

*Encourages Active Learning **.*** A common criticism of didactic lecture formats is the tendency for the learning process to devolve into a monotonous process of transferring facts, followed by a reliance on the rote memorization of those facts ( White, 2011). Many educators have found that learning outcomes are improved when students are engaged in ‘active learning’ ( Prince, 2004; Handelsman *et al.,* 2004; Miller and Metz, 2014). Active learning is the process of ongoing and adaptive dialogue between teacher and student ( Laurillard, 2002). In such an environment, active learning gives students time to reflect on their understanding of recent material, practice a skill or highlight gaps in their knowledge before engaging in class discussion ( Prince, 2004). Active learning encourages students to accomplish higher order objectives on Bloom’s taxonomy, such as analysis, synthesis and evaluation. ( Bloom, 1956) Many studies have shown that active learning processes can improve student’s comprehension, problem-solving abilities and critical thinking skills ( Michael, 2006; Bonwell and Eison, 1991; McKeachie, 1994; Palmer and Devitt, 2007).

*Improves Student Attendance **.*** Many studies on ARS have shown that there is an increase in class attendance when the use of ARS is tied to class grading ( Kay and Lesage, 2009). Such motivation to attend class has been observed even when the attendance represents as little as 5% of the grade ( Caldwell, 2007). In three studies, improved class attendance was also observed when grades were not associated with ARS use ( El-Rady, 2006; Preszler *et al.,* 2007), including that of a Pediatric Residency training program at the Mayo College of Medicine ( Homme, Asay and Morgenstern, 2004).

### The Cons

Six distinct categories of drawbacks were noted in the compilation of the research studies (see Table 2). These are discussed, in turn, below.

*Teacher Prep Time.* The use of ARS in the classroom requires a concerted effort on the part of the teacher to prepare this part of the lecture ( Collins, 2008). Failure in this area by the teacher can have the resulting effect of student disenchantment and demotivation ( Abate, Gomes and Linton, 2011; Ioannou and Artino, 2007; Kay and Lesage, 2009; Duggan, Palmer and Devitt, 2007). Some teachers found the preparation for the questions time-consuming, particularly because the questions to be posed must be well formulated ( Abate, Gomes and Linton, 2011; Arneja *et al.,* 2009; Connor, 2009; Duggan, Palmer and Devitt, 2007; Gauci *et al.,* 2009; Bruff, 2018). Well formulated questions elucidate the student’s true level of understanding, are not too simple and boring, and are not too complex or confusing. Allen and Tanner (2005) noted that “multiple choice questions are a difficult format in which to pose questions that nudge students to the realm of higher order thinking.” The answer records, using a multiple choice questioning approach, “do not provide explanations of the reasons or reasoning patterns for students’ selection.”

*Cost.*Audience Response Systems are not free. System cost varies considerably but, in the end, the costs must be borne either by the institution, the student or both and this can be a deterrent ( Cain and Robinson, 2008; Mastoridis and Kladidas, 2010; Menon *et al.,* 2004). Wood (2004) reported that clickers cost between $5 - $30 while the audience response system itself costs about $1000 for each 100 students. Barber and Njus (2007) reported a net cost to the University bookstore of $16 for the clicker and a per term charge of between $13 - $15 per student.

*Learning to Use.* Utilizing the clickers as a student essentially requires no training. The institution, on the other hand, has to install the hardware and software and ensure proper network connectivity to all the devices ( Barber and Njus, 2007). The teacher, for their part, needs to learn how to integrate the system into the lecture format of the computer system ( Arneja *et al.,* 2009; Cain and Robinson, 2008; Kay and Lesage, 2009).

*Technical Malfunctions.* ARS are wireless electronic devices working on a software platform that can be subject to periodic malfunctions ( Benson *et al.,* 2016). Interruptions in wireless signals, glitches in software, or mechanical breakage in the clickers can render the system inoperable. Such events, however rare, require the attention of a staff technician.

*Reduced Lecture Time.* In several studies ( Abate, Gomes and Linton, 2011; Connor, 2009; Ioannou and Artino, 2007; Kay and Lesage, 2009), it was noted that the reduced time for lecture is a negative attribute of the ARS approach. At the very least, the use of ARS requires some adaptation to the lecture format to cover less lecture material and make room for questions. This is not always a welcomed feature by all instructors.

*Students Do Not Want to be Monitored.* Two of the features of ARS are the ability to use the technology to track student attendance and record answers to the multiple choice questions asked during class. One study ( Kay and LeSage, 2009) noted that students were displeased about being forced to attend lectures in order to earn academic credit for a class. In other studies ( Caldwell, 2007; Fujikura *et al.,* 2013) it was found that students disapproved of grades being attached to ARS participation. Caldwell (2007) noted that “Attaching a grade to ARS may undermine the goal of developing an effective learning environment.” Meedzan and Fisher (2009) also found that there was lower satisfaction to class participation when ARS was used as a tool for summative assessment.

## Conclusion

A review of the literature has demonstrated that Audience Response Systems (ARS) have been widely employed at all levels in the education of health professionals and that such systems have clear benefits and drawbacks from both the perspective of the students and the teachers. Benefits were found to fit ten (10) distinct categories while drawbacks fit into six (6) discrete categories. ARS can provide a complementary pedagogical approach to the traditional didactic lecture format and provide exciting potential for improving the education of health professionals in areas such as improved learning outcomes, greater class attendance, and encouraged active learning.

## Take Home Messages

• Audience Response Systems (ARS) have been widely employed at all levels in the education of health professionals
• ARS can provide a complementary pedagogical approach to traditional didactic lecture formats
• Experiences with the use of ARS, by both students and teachers, has led to the identification of a distinct set of benefits and drawbacks
• ARS has exciting potential for improving the education of health professionals in areas such as improved learning outcomes, greater class attendance, and encouraged active learning

## Notes On Contributors

**Ryan Denkewicz** BS BioMedical Engineer is a 4th year Medical student at the Frank H. Netter School of Medicine at Quinnipiac University, North Haven, Connecticut. His academic interests include understanding the means by which students are motivated to learn. ORCID ID https://orcid.org/0000-0002-5683-5803

## Declarations

The author has declared that there are no conflicts of interest.

## Ethics Statement

An ethics review was not required as this is a literature review only.

## External Funding

This article has not had any External Funding
