# Supplementary material for: Pros and Cons of Audience Response Systems in the Education of Health Professionals
Source: MedEdPublish (2016). 2019 Sep 24;8:182. [Version 1] doi: 10.15694/mep.2019.000182.1 (PMC10712486; doi:10.15694/mep.2019.000182.1)
Supplement: Peer review history

# Reviewer report: Reviewer response for version 1

**DOI:** 10.21956/mep.19918.r30188

**Published:** 2019-12-08

**Authors:** P Ravi Shankar

**Affiliations:** 1 American International Medical University

This review has been migrated. The reviewer awarded 4 stars out of 5 Audience response system (ARS) is being increasingly used in health science education. This is a comprehensive review of the benefits and drawbacks of ARS. A more recent development is the possibility of using ARS using cell phones and tablets without the need for clickers and the hardware set up. This opens up the possibility of minimizing the cost and widening the use of the technology. I had used the technology (using traditional clickers) at Aruba and we encountered a number of difficulties in using the system. I am recently working on using ARS with cell phones and tablets and free polling software. This article would be of interest to all medical educators.

Reviewer Expertise:

NA

No decision status is available

# Reviewer report: Reviewer response for version 1

**DOI:** 10.21956/mep.19918.r30190

**Published:** 2019-10-05

**Authors:** Lubna Jahanzeb

**Affiliations:** 1 Baqqai Medical University

This review has been migrated. The reviewer awarded 4 stars out of 5 A very detailed and informative review. It explains the pros and cos in a very deep, insightful and interesting manner. Each point in this article is supported by a strong reference which increases the worth of paper. Moreover, it is great to see students working in this important area.

Reviewer Expertise:

NA

No decision status is available

# Reviewer report: Reviewer response for version 1

**DOI:** 10.21956/mep.19918.r30189

**Published:** 2019-09-27

**Authors:** BALAJI ARUMUGAM

**Affiliations:** 1 TAGORE MEDICAL COLLEGE AND HOSPITAL

This review has been migrated. The reviewer awarded 4 stars out of 5 A review of 120 articles on audience response system and narrating the pros and cons of the ARS was very clearly mentioned and the study looks comprehensive. Actually its a great effort by the authors to publish such a narrative review on ARS. The various names of the ARS was mentioned in the introduction which was quoted with references was amazing. These studies are still a tough to conduct and get the feedback from the students in the resource poor settings. The advanced technology and the methods of getting ARS was nicely narrated in the study. The listed pros and cons with various studies demonstrating with the statistical significance. The cited evidence of improved learning outcomes presented from the recent studies of 2005 to 2015. Overall this study will be very useful for the faculty those who are working on this area of interest.

Reviewer Expertise:

NA

No decision status is available

# Reviewer report: Reviewer response for version 1

**DOI:** 10.21956/mep.19918.r30187

**Published:** 2019-09-24

**Authors:** Julie Hunt

**Affiliations:** 1 Lincoln Memorial University

This review has been migrated. The reviewer awarded 3 stars out of 5 This review paper summarizes the basic pros/cons of using audience response devices, or "clickers", in lecture-based pre-medical and medical education. The author does a good job of assembling diverse literature into themes and correctly identifies many of the benefits and the relatively few drawbacks of using these devices in medical education. I think the article is helpful for educators who may be considering implementing the use of ARS devices in their lectures.The article could have benefited from having more details on the literature search criteria used, including the dates of articles searched and how articles were determined to be included or excluded. Also, although the article described the use of multiple choice questions embedded in lectures--the most common use of ARS devices in education---it missed mentioning the many other uses of these devices, which include uses such as providing free text answers or survey data to guide how instructors cover topics, or using the self-paced poling feature to allow students to submit answers to quizzes or tests with instant data capture.

Reviewer Expertise:

NA

No decision status is available
